# The comparison of the three assays for determination of fecal calprotectin in inflammatory bowel disease

**DOI:** 10.11613/BM.2021.020707

**Published:** 2021-04-15

**Authors:** Joško Osredkar, Tina Kurent, Teja Fabjan, Kristina Kumer, Elizabeta Božnar Alič, David Drobne

**Affiliations:** 1 Institute of Clinical Chemistry and Biochemistry, University Medical Centre Ljubljana, Ljubljana, Slovenia; 2 Faculty of Pharmacy, University of Ljubljana, Ljubljana, Slovenia; 3 Department of Gastroenterology, University Medical Centre Ljubljana, Ljubljana, Slovenia; 4 Medical Faculty, University of Ljubljana, Ljubljana, Slovenia

**Keywords:** inflammatory bowel disease, feces, biomarkers, leukocyte L1 antigen complex

## Abstract

**Introduction:**

Fecal calprotectin is a biomarker for monitoring inflammatory bowel disease (IBD) activity. Our aim, therefore, was to evaluate two new assays, the point of care test Quantum Blue and the Liaison Calprotectin with respect to the Calprest, commonly used assay, and to determine their performance for IBD diagnosis.

**Materials and methods:**

We included 73 prospective patients with IBD. Fecal calprotectin was measured and analysed with the routine Calprest assay and two recently introduced assays, the Quantum Blue and the Liaison Calprotectin. Furthermore, we compared the results by Bland and Altman analysis, and Passing-Bablok regression.

**Results:**

We observed no difference in median calprotectin values obtained by the Calprest (94.6 µg/g, 95%CI 66.5 to 166.1) and Liaison assay (101.0 µg/g, 95%CI 48.1 to 180.1) whereas significantly higher concentrations were obtained with the Quantum Blue assay (240.0 µg/g, 95%CI 119.9 to 353.2). The mean absolute and relative difference between the Calprest and Quantum Blue methods was statistically significant (- 162.3 µg/g and - 143.1%). Mean absolute difference between the Calprest and Liaison calprotectin methods was positive (2.2 µg/g). The agreement between assays revealed that Quantum Blue and Calprest have fair agreement with Kappa coefficient of 0.38 (95%CI 0.26 to 0.51). Liaison Calprotectin and Calprest revealed moderate agreement with a weak Kappa coefficient of 0.47 (95%CI 0.32 to 0.62).

**Conclusion:**

Clinicians should be aware of these differences between the assays and avoid comparison of their respective results.

## Introduction

Inflammatory bowel disease (IBD) is a chronic inflammatory condition of the intestine, characterized by bloody diarrhea, anorexia and fever. Left untreated, it leads to severe complications, resulting in numerous surgeries and eventually intestinal failure. The disease is treated with anti-inflammatory drugs, such as local and systemic steroids, thiopurines and inhibitors of different cytokines, *e.g.* tumor-necrosis factor alpha and interleukins (IL-6 and IL-12) (*1*, *2*). An important objective of anti-inflammatory treatment is also biochemical remission, revealed by normalization of inflammatory markers, such as C-reactive protein and fecal calprotectin (*3*-*6*). The latter is currently recognized as the most sensitive biomarker for monitoring inflammatory activity of this disease. Calprotectin is a calcium- and zinc-binding cytosolic protein, a S100A8/A9 heterodimer of molecular weight 36.5 kDa, which is mainly released by neutrophils and to a lesser extent by monocytes and epithelial cells. Its discharge stems from cell disruption and apoptosis in the intestinal lumen, becoming detectable when flushed away with feces. The fecal calprotectin concentration thus reflects the degree of pathological neutrophilic infiltration of the intestinal wall (*7*). Since this is a non-invasive test, it gained popularity in clinical practice for monitoring disease activity and adapting anti-inflammatory treatment. Consequently, many different non-invasive fecal calprotectin assays for its determination have been developed in recent years, yet inadequately tested against the commonly used test in hospital laboratories, the Calprest enzyme linked immunosorbent assay (ELISA).

The aim of this study was to help clinicians in their decision-making processes when using different laboratory results due to different tests which also have different cut-off values. In this study, we compared the performance of two recently introduced assays, the point of care test (POCT) Quantum Blue (Bühlmann, Schonenbuch, Switzerland) and Liaison calprotectin assay (Diasorin, Saluggia, Italy), against that of the commonly used test Calprest assay (Eurospital, Trieste, Italy). Calprotectin is often measured with ELISA. The ELISA is time consuming and mostly suited for analysing samples in batch. Compared to the ELISA method Quantum Blue and Liaison methods are faster, cheaper, with easier sample preparation and allow real-time more accessible analysis.

## Materials and methods

This study was performed at the University Medical Centre Ljubljana (UMCL), Slovenia. Stool samples were collected in the period from 2014 to 2016.

We included 73 consecutive patients treated at the tertiary referral centre with established diagnosis of IBD. No relevant exclusion criteria were used. The diagnosis of IBD was based on clinical, biochemical and endoscopic criteria in line with the European Crohn’s and Colitis guidelines (*8*). Fecal calprotectin was determined by the three concerned assays in each fecal sample. All patients signed an informed consent. The research proposal was approved at the 129^th^ session of the UMCL Expert Council, which was held on 23^rd^ April 2014.

### Calprotectin measurements

For analytical performances we used three quantitative assays (one of them POCT) in question, with specifications listed in Table 1. To assess the agreement among the three assays we used the medical decision cut-off of < 50 µg/g for calprotectin concentrations as negative.

**Table 1:** Assay characteristics

**Assay (manufacturer)**	**Method**	**Extraction device**	**Measurement range** **(µg/g)**	**Cut off ** **(µg/g)**
Calprest (Eurospital, Trieste, Italy)	ELISA	Eurospital extraction device	15.6 - 500	70
Liaison Calprotectin kit (Diasorin, Saluggia, Italy)	CLIA	Diasorin extraction device	5 - 800	50
Quantum Blue (Bühlmann, Schonenbuch, Switzerland)	Quantitative immunochromatography	Bühlmann fecal extraction device	30 - 1000	50
ELISA - enzyme-linked immunosorbent assay. CLIA - chemiluminescence immunoassay.				

This cut-off was selected based on our experience and published data as it correlates with clinically meaningful outcomes such as endoscopic improvement in both, ulcerative colitis and Crohn’s disease (*9*-*11*).

To prevent preanalytical variation, all samples were extracted in accordance with the manufacturers’ instructions, using specific fecal calprotectin buffers of manufactures. Details of extraction technique for each assay are given below. We used manufacturer calibration and control samples. Control samples were analysed before patient’s samples. We used two levels of Bühlmann control samples (B-CALE-CONSET), low with expected range 1 - 30 µg/g and high with expected range 197 – 592 µg/g. The point-of-care Quantum Blue Reader was adjusted with white RFID chip card in order to change lot specific test parameters. For Liaison we used Calprotectin control set, Control 1 with expected range: 40.7 – 71.2 µg/g and Control 2 with expected range: 187 – 333 µg/g. For calibration we used manufacturer calibrators (Liaison Calibrator 1 and Calibrator 2). For Calprest we used two level control samples (Control 1 and Control 2) and six standards for calibration curve. Comparison included assessment for separation of active *vs.* quiescent disease with the currently accepted cut-off of 50 µg/g. The diagnosis of IBD was based on clinical, biochemical and endoscopic criteria in line with the European Crohn’s and Colitis guidelines (*8*).

Fresh stool samples brought to the clinic by the patients were divided into two parts. One sample was used for routine analysis with Calprest and the other sample was frozen (- 20 °C) until the analysis by the other two methods. For Calprest immunoassay samples were stored in a refrigerator (2-8 °C) and analysed once a week as provided in the routine protocol. For Liaison and Buhlmann samples were stored frozen for up to 6 months. Upon thawing, extracts of stool samples were simultaneously prepared and measured calprotectin levels with both immunoassays in the same day.

### Quantum Blue, Bühlmann

This POCT assay is based on a quantitative sandwich lateral immunochromatography assay. In our study, we used the extended range (30-1000 μg/g) cartridges. Fecal samples were prepared with Bühlmann Smart Prep device and measurements performed according to the manufacturer’s instructions. Briefly, stool extract was diluted 1:10 with extraction buffer, mixed and centrifuged for 5 min at 3000xg. Supernatant (60 µl) was loaded onto the sample loading port of the cartridge. This cartridge was then read by a Quantum Blue reader (*12*). All samples were analysed in one batch.

### Liaison Calprotectin, Diasorin

Samples were prepared using the Liaison Calprotectin Stool Extraction Device. After stool sample collection, 6 ml of extraction buffer was added and following homogenization on a multi-tube vortex for 30 min, the analysis was performed by Liaison analyser. This analyser uses a chemiluminescent immunoassay for the quantitative determination of calprotectin. The measurements were performed in one batch. Calibrators, controls and samples were analysed according to the manufacturer`s instructions (*13*).

### Calprest, Eurospital

Calprest is an enzyme immunoassay where polyclonal antibodies react with calprotectin in extracted stool sample. After stool sample collection, a part was reintroduced into the tube containing the 2.5 mL of extraction solution. Each tube was vortexed for 60 seconds in order to properly homogenate the content, placed on a roller shaker and shaken for 60 minutes. Before the analysis by an ELISA sample processor the sample was centrifuged one more time to remove the possible residuals of fecal material and diluted 1:50 (*14*).

### Statistical analysis

Statistical analysis was performed using MedCalc Software version 12.0 (MedCalc Software Ltd, Ostend, Belgium). We used the Kolmogorow-Smirnov test to assess the normality of data. The agreement between three different assays was determined by Bland and Altman plot and Passing-Bablok analysis. The Cohen’s kappa statistics was used to estimate Inter-rater reliability. Only those results within the measurement range of the assay were included. P-value < 0.05 was considered significant.

## Results

Samples from 73 patients were collected. For calculation we used the calprotectin results that were in the linear range with all three methods. Therefore, we took into account only 37 patients with the median age of 41 years (19-74).

We observed no difference in median calprotectin values obtained by the Calprest (94.6 µg/g, 95%Cl 66.5 - 166.1) and Liaison assay (median 101.0 µg/g, 95%Cl 48.1-180.1), whereas median values were significantly higher with the Quantum Blue assay (240.0 µg/g, 95%Cl 119.9 - 353.2).

**Figure 1:**
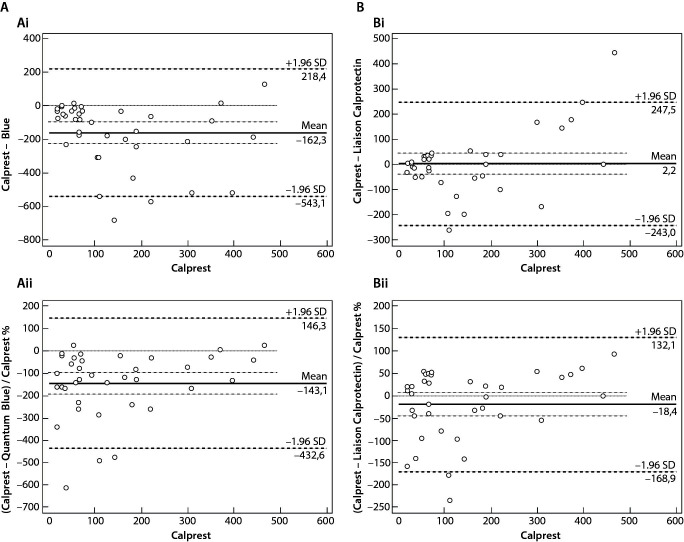
Absolute and relative Bland and Altman plot with the presentation of the limits of agreement. A: Comparison between the Calprest and Quantum Blue (mean difference = - 162.3 with 95%Cl of - 227.1 to - 97.6). B: Comparison between the Calprest and Calprotectin Liaison (mean difference = 2.22 with 95%Cl of - 39.5 to – 43.9).

As shown in Figure 1 Ai the mean absolute difference between the Calprest and Quantum Blue methods was negative - 162.3 μg/g with 95%Cl of - 227.1 μg/g to - 97.6 μg/g, and an agreement range from - 543.1 μg/g to 218.4 μg/g. As shown in Figure 1Aii the relative difference was -143.1% with 95%Cl of - 192.4% to - 93.9 and agreement limits are from - 432.6% to 146.3%. The difference is statistically significant and show negative trend, proportional to the magnitude of the measurement. The bias seems to becoming lower when the concentration is higher. The values outside the 95% confidence interval emerged mainly in samples with high calprotectin concentrations. Moreover the regression equation y = - 36.6 + 2.4x with 95%Cl for intercept - 118.19 to 27.27 and for slope 1.75 to 3.50 showed the same findings. These two methods can not be used simultaneously and interchangeably.

**Figure 2:**
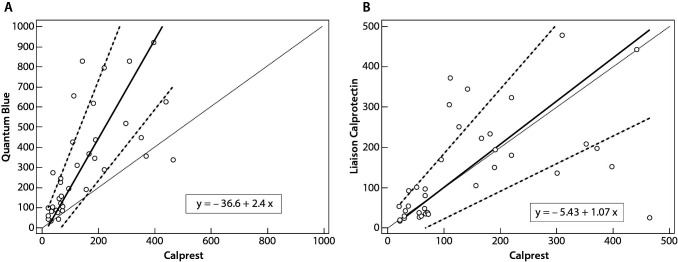
A: Regression analysis of the Calprest and Quantum Blue. Regression line equation (solid line): y = - 36.6 + 2.4x; 95%Cl (dashed line) for intercept - 118.19 to 27.27 and for slope 1.75 to 3.50. B: Regression analysis of the Calprest and Liaison Calprotectin. Regression line equation (solid line): y = - 5.43 + 1.07x; 95%Cl (dashed line) for intercept - 44.86 to 21.32 and for slope 0.68 to 1.60. Cunsum test for linearity indicates no significant deviations from linearity (P > 0.10). The dotted line is the equality line.

As shown in Figure 1Bi the mean absolute difference between the Calprest and Liaison calprotectin methods was positive 2.2 μg/g with 95%Cl of - 39.5 μg/g to 43.9 μg/g and an agreement range from - 243.0 μg/g to 247.5 μg/g. As shown in Figure 1Bii the relative difference was - 18.4% with 95% Cl of - 44.0% to 7.2 and agreement limits are from 87.9% to 176.3%. The difference is not statistically significant and show positive trend, proportional to the magnitude of the measurement. The bias seems to becoming lower when the concentration is higher. The difference between these two methods is small and can be used simultaneously and interchangeably. Moreover the regression equation y = - 5.43 + 1.07x with 95%Cl for intercept - 44.86 to 21.32 and for slope 0.68 to 1.60 showed the same findings (Figure 2). These two methods can be used simultaneously and interchangeably.

Between the Calprest and Liason Calprotectin methods, we obtained - 18.4% bias, and between the Calprest and Quantum Blue, - 143.1%. Only bias between Calprest and Quantum Blue are lower than the allowable bias: 22.6% for measuring calprotectin concentration taken by Juricic *et al* (*15*).

The agreement between assays revealed that Quantum Blue and Calprest have fair agreement with Kappa coefficient of 0.38 (95%Cl 0.26 to 0.51). Liaison Calprotectin and Calprest revealed moderate agreement with a weak Kappa coefficient of 0.47 (95%Cl 0.32 to 0.62).

## Discussion

Our finding was that the higher concentrations of calprotectin were measured using the Quantum Blue when compared to Liaison and Calprest. However, regression analysis confirmed significant differences between the Quantum Blue and Calprest, even though manufacturers propose identical cut-offs, indicating that comparison of absolute values between different assays is inappropriate, an issue highlighted previously also by others (*16*, *17*). The differences could account to the commercial assays, which have antibodies targeting different calprotectin epitopes, or have different extraction device. Even though previous studies have shown the differences between the extractions kits and the weight method, overall, the clinical interpretation of the results does not change.

Differences in preanalytical manipulation of samples with different assays might have influenced our results as reported previously (*15*). This further stresses the need for universal fecal calprotectin standard. Promising reports were published recently by Nilsen *et al*, who had prepared a purified calprotectin antigen from human granulocytes. Such a standard could serve as international calibrator in the future (*18*).

In this study, we focused on patients with IBD. For this reason, our findings should not be generalized to patients with irritable bowel disease where the usual clinical dilemma, based on fecal calprotectin assay, is whether to proceed to invasive diagnostics. However, since we did not have endoscopic golden standard, we cannot exclude that. But as patients with new diagnosis of inflammatory bowel disease generally possess very high values of fecal calprotectin, we can speculate that all three assays would perform equally well in this clinical setting.

A provocative question concerning inflammatory bowel disease is the cut-off of fecal calprotectin that should be targeted with anti-inflammatory drugs. Fecal calprotectin as such is not considered a treatment target, but rather an adjunct objective as a surrogate of mucosal healing (*6*). Generally, target fecal calprotectin concentrations should be lower than 100 - 250 µg/g (*9*, *19*, *20*). Recently, important piece of evidence has been obtained for using fecal calprotectin for treatment optimization in Crohn’s disease, as patients monitored with serial fecal calprotectin had significantly better clinical and endoscopic outcomes compared to those followed only clinically (*21*). In this trial, fecal calprotectin concentration of 250 µg/g was used to trigger treatment escalation. However, clinicians should be careful here, because different calprotectin assays give different numerical values, which is also confirmed in our study. However, it is reassuring that higher biases of calprotectin assays were observed mainly with higher values in our study. Therefore, most assays can still be reliably used for treatment optimization which typically relies on lower cut-offs (50 -150 µg/g).

When we compare our results with similar studies, we see that other authors got comparable results. Goll *et al.* in their study found good correlation between assays, however a non-linear difference was found: at values below 200 mg/kg, no significant bias was found; in the interval 200 - 1000 mg/kg the Calprest assay measured on average 30% lower than Calpro; and at higher values Calprest measured 60% higher values than Calpro (*22*). Haisma *et al.* evaluated by how much different tests differed from the trusted ELISA method, and found that in the lower ranges the difference was small enough not to cause problems in interpretation. They concluded that in order to minimize wrongful interpretation of calprotectin changes over time it is essential to always use the same test of the same manufacturer (*23*). We are aware that our study has shortcomings. Above all, we have a relatively small number of patients, and without the control group. With a larger number of patients, another aspect could be taken into account, *i.e.* the duration of the disease itself and the onset of the disease in an individual patient.

The results of the study confirmed that in addition to standard ELISA methods for monitoring calprotectin concentrations in patients with chronic inflammatory bowel disease, Quantum Blue and Liaison calprotectin methods are sufficiently reliable. Compared to the ELISA method are faster, cheaper, with easier sample preparation and allow real-time more accessible sample analysis, which is extremely important for continuous monitoring the dynamics and success of treatment of this disease.

To conclude, in this study we compared three different assays for determination of fecal calprotectin. Quantum blue yields higher values compared to Calprest and Liaison. Clinicians should be aware of these differences when interpreting the results of different assays in clinical practice and when interpreting the findings in scientific reports.
